# Traditional Chinese Medicine Syndromes are Associated with Driver Gene Mutations and Clinical Characteristics in Patients with Lung Adenocarcinoma

**DOI:** 10.1155/2022/9905868

**Published:** 2022-10-07

**Authors:** Jili Yang, Haiyan Lu, Niancai Jing, Bo Wang, Huanyu Guo, Shoukun Sun, Yue Zhang

**Affiliations:** ^1^Department of Integrative Medicine, Jilin Provincial Cancer Hospital, Changchun 130012, China; ^2^Changchun University of Traditional Chinese Medicine, Changchun 130117, China

## Abstract

This study aimed to investigate the associations between traditional Chinese medicine (TCM) syndromes and driver gene mutations as well as the clinical characteristics of patients with lung adenocarcinoma. We performed a cross-sectional study in patients with lung adenocarcinoma between June 2020 and October 2021. The patient characteristics, such as age, sex, smoking history, clinical stage, metastasis, driver gene mutations, and the type of traditional Chinese medicine syndrome/element, were collected. The associations between each TCM syndrome and sex, smoking history, clinical stage, metastasis, and driver gene mutations were analyzed. The present study included 127 patients. The most frequent TCM syndromes were Qi and Yin deficiency (39, 30.7%) and lung-spleen Qi deficiency (32, 25.2%). Eighty-one (63.8%) patients had mutations in driver genes, especially in the *EGFR* gene (64, 79.0%). There was a statistically significant association between a driver gene mutation and TCM syndrome (*P* < 0.05). Genetic mutations presented more frequently in patients with Qi and Yin deficiency (37.0%), lung-spleen Qi deficiency (30.0%), or the cold element (59.3%). Male patients were more likely to have Qi stagnation and blood stasis, whereas female patients were more likely to have lung-spleen Qi deficiency or Qi and Yin deficiency. The patients with lung-spleen Qi deficiency were usually younger than those with Qi and Yin deficiency or Qi stagnation and blood stasis (*P* < 0.05). Compared with the patients with other TCM syndromes, the patients with Yin and Yang deficiency were more likely to have bone metastasis. TCM syndromes were associated with driver gene mutations, sex, age, and bone metastasis in patients with lung adenocarcinoma.

## 1. Introduction

Lung cancer is the second most common cancer and the leading cause of cancer mortality worldwide [1]. Adenocarcinoma is the most common type of primary lung cancer [2]. Its main treatments include surgical resection, chemoradiation, and immunotherapy [3]. In addition, traditional Chinese medicine (TCM), as one of the most commonly used alternative and complementary medicine approaches, has been applied as an adjuvant therapy to improve the survival and quality of life of patients with lung cancer [4–6].

With advances of molecular genetics technology, an increasing number of genetic mutations have been identified in patients with lung cancer [7]. This has led to the development of genotype-directed targeted therapy [8]. For example, mutations in a special group of genes, called driver genes, have been found to be causally associated with lung cancer development and growth and may have therapeutic indications [9]. The epidermal growth factor receptor (EGFR) is a transmembrane protein responsible for cell growth. Its gene carries the most frequently identified driver mutations in Asian patients with lung adenocarcinoma [10, 11]. Studies have found that personalized treatments with genotype-directed therapies targeted to lung cancer patients with *EGFR* mutations can significantly improve their survival and quality of life [12, 13]. Recent studies have further demonstrated that TCM combined with *EGFR* mutation-targeted therapy provides better efficacy than the latter therapy alone in patients with lung cancer [14, 15]. TCM diagnosis and therapy are based on syndrome differentiation. The TCM syndrome name summarizes the disease etiology, location, nature, and status at a certain stage of disease development. The TCM element is one of the basic components of each TCM syndrome. It determines the disease's nature as cold, heat, deficiency, excess, or mixed deficiency and excess. Experienced TCM physicians can select different treatment formulas to individualize acupuncture or herbal treatments based on different TCM syndromes and elements [16]. However, whether TCM may be an individualized treatment to target lung cancer patients with different driver gene mutations is unknown. The first step to answer this question is to study the potential associations between TCM syndromes and driver gene mutations in lung cancer patients.

Therefore, this study aimed to investigate the associations between TCM syndromes and driver gene mutations, as well as the clinical characteristics of patients with lung adenocarcinoma. We hope that our study results provide clinical evidence for the development of personalized TCM treatment options for these patients.

## 2. Materials and Methods

### 2.1. Study Design and Participant Selection

We performed a cross-sectional study in patients with lung adenocarcinoma at Jilin Cancer Hospital, Jilin, China, between June 2020 and October 2021. The study protocol was approved by the hospital's ethics committee (approval number: 202011-41-01). All of the study participants signed an informed consent form.

The inclusion criteria were as follows: (1) age >18 years old; (2) primary lung adenocarcinoma diagnosis based on a pathological examination; (3) without any previous treatment (including TCM) for lung cancer; (4) consented to genetic testing. The exclusion criteria were as follows: (1) with other types of cancer or cancer metastasis to the lung; (2) with severe heart, liver, brain, or kidney disease; (3) unable to sign the informed consent form.

### 2.2. Study Protocol

After the informed consent process, the age, sex, and smoking history of the patients were documented. Diagnosis, pathological classification, clinical stage, and metastasis were determined based on the clinical evaluations, according to published guidelines [17, 18]. TCM syndrome differentiations were based on the method proposed by Liu et al. 19, 20. Two experienced TCM attending physicians evaluated the enrolled patients separately and determined the type of TCM syndrome. Any discrepancy between them was discussed, and a third TCM attending physician was consulted to make the final syndrome determination. There were five types of TCM syndromes, including lung-spleen Qi deficiency, Yin deficiency and internal heat, Qi and Yin deficiency, Qi stagnation and blood stasis, and Yin and Yang deficiency. TCM syndromes were also classified into five elements, including cold, heat, deficiency, excess, and mixed deficiency and excess.

Genetic mutation analysis of driver genes, including *EGFR*, *ALK*, *ROS1*, *KRAS*, *BRAF*, *ERBB2 (HER-2)*, *RET*, *MET*, and *NTRK*, was performed in the Pathology Department of Jilin Cancer Hospital. The laboratory methods used in this study were the same as those described previously [21]. Briefly, second-generation genetic detection technology was used. The tissue sample was extracted by using a QIAamp DNA FFPE tissue extraction kit (Qiagen, Germantown, MD, USA). The DNA sequencing library was constructed using an Illumina TruSeq kit (Illumina, San Diego, CA, USA) and subjected for sequencing using the Illumina HiSeq 4000 sequencing platform (Illumina), with a read length of PE150 and a sequencing depth of >500×. The obtained raw data were filtered for bioinformatics analysis.

### 2.3. Statistical Analysis

Continuous data are presented as the mean ± standard deviation and were analyzed by the *t*-test or one-way analysis of variance. Categorical data are presented as a number with frequency and were analyzed by the chi-squared test or Fisher's exact test. All statistical analyses were performed by using SPSS (version 25.0, SPSS, IBM, New York, USA). A *P*value < 0.05 was considered statistically significant.

## 3. Results and Discussion

### 3.1. Characteristics of Study Participants

A total of 127 patients were included in this study, with 59 male and 68 female patients. The mean age was 63.5 years (range: 39–81 years old). A total of 56 patients had a history of smoking. In addition, there were 8 (6.3%), 18 (14.2%), and 101 (79.5%) patients in clinical stage I, II, III, and IV, respectively. TCM syndrome differentiations resulted in 32 (25.2%), 29 (22.8%), 39 (30.7%), 19 (15.0%), and 8 (6.3%) patients with lung-spleen Qi deficiency, Yin deficiency and internal heat, Qi and Yin deficiency, Qi stagnation and blood stasis, and Yin and Yang deficiency, respectively. In addition, 66 (52.0%), 61 (48.0%), 72 (56.7%), 29 (22.8%), and 26 (20.5%) patients presented with the elements of cold, heat, deficiency, excess, and mixed deficiency and excess, respectively.

Driver gene mutations were identified in 81 (63.8%) patients. Most of them (64, 79.0%) were mutations or deletions in the *EGFR* gene, with the exon 19 deletion and the L858R mutation in exon 21 as the most frequent genetic changes (33, 40.7% and 24, 29.6%, respectively). Other mutations included 6 (7.4%) patients with an ALK mutation, 3 (3.7%) patients with RET and KRAS mutations, 1 (1.2%) patient with BRAF V600E, MET exon 14 skipping mutation, and ERBB-2 mutations, 2 (2.5%) patients with other mutations, and 8 (9.9%) patients with compound mutations. The clinical characteristics of the patients with and without driver gene mutations are shown in Table 1. Compared with the patients with no driver gene mutations, the patients with driver gene mutations were statistically significantly more likely to have a history of smoking. Otherwise, there were no statistically significant differences in age, sex distribution, clinical stage, or metastasis status between the two patient groups.

### 3.2. Associations between TCM Syndromes and Driver Gene Mutations

As shown in Table 2, there were statistically significant associations between TCM syndromes and driver gene mutations. Most driver gene mutations were identified in the patients with the TCM syndrome of Qi and Yin deficiency or lung-spleen Qi deficiency. Most patients with Yin and Yang deficiency carried wild-type driver genes.

In terms of the TCM syndrome element, driver gene mutations had statistically significant associations with the TCM elements of cold and heat, but not with the elements of deficiency and excess. Most driver gene mutations were identified in patients with the TCM syndrome element of cold or deficiency (Table 3). The distributions of different TCM syndromes and elements in patients with or without driver gene mutations are shown in Table 4. In patients with genetic mutations who had the syndrome of Qi and Yin deficiency or lung-spleen Qi deficiency, the most common elements were cold (23 (95.8%) and 22 (73.3%), respectively) and deficiency (16 (66.7%) and 21 (70.0%), respectively).

### 3.3. Associations between TCM Syndromes and Clinical Characteristics

As shown in Table 5, there were significant differences in the sex distribution in patients with lung-spleen Qi deficiency, Qi and Yin deficiency, or Qi stagnation and blood stasis. The female patients more often had lung-spleen Qi deficiency or Qi and Yin deficiency, whereas the male patients more frequently had Qi stagnation and blood stasis. Most of the patients reported no history of smoking and had clinical stage IV lung cancer. There were no statistically significant associations between TCM syndromes and the smoking history or clinical stage.  Table 6 shows the mean ages of patients with various TCM syndromes, with the youngest age seen in patients with lung-spleen Qi deficiency and the oldest age seen in patients with Qi and Yin deficiency. There were statistically significant differences in age between the patients with lung-spleen deficiency and those with Qi and Yin deficiency, as well as between the patients with lung-spleen deficiency and those with Qi stagnation and blood stasis.

### 3.4. Association between TCM Syndromes and Cancer Metastasis

Lymph nodes were the most common location of metastasis. TCM syndromes were associated with bone metastasis but not with other metastases. Compared with the patients with other TCM syndromes, the patients with Yin and Yang deficiency were more likely to have bone metastasis (Table 7).

## 4. Discussion

Driver gene mutations are common in patients with lung adenocarcinoma. In addition, genotype-directed therapies targeted to these driver genes have shown promising outcomes in clinical practice. TCM also can be used as an adjuvant therapy to treat patients with lung cancer. The present study showed that driver gene mutations as well as age, sex, and bone metastasis were associated with TCM syndromes in patients with lung adenocarcinoma. Our results provide a foundation for further research toward an individualized genotype-directed TCM therapy in these patients.

In TCM, lung cancer was known as “lung retention” and “lung amassment” in ancient times. The pathogenesis of lung cancer is believed to be due to deficiency, phlegm, stasis, and toxins. Cancer toxin is an independent pathogenic factor of lung cancer. Most patients with lung cancer suffer from vital Qi deficiency, dysfunction of Zang-fu, pathogenic toxin attacks following Qi deficiency, lung Qi stagnation, dysregulated water pathway, undistributed fluid, phlegm accumulation from fluid, Qi stagnation and phlegm coagulation, impediments of blood vessels, internal obstruction of static blood, phlegm retention, and toxin accumulation, which finally results in a mass in the lung over time. As stated in the “Essential Reading of Chinese Medicine Principles,” “the retention is caused by the deficiency of vital Qi and the excessive accumulation of pathogenic Qi.”

Even though driver gene mutations have been extensively studied in lung cancer research, there are few reports on the associations between driver gene mutations and TCM syndromes or cold and heat elements. As stated in The Yellow Emperor's Classic of Medicine, “when Yin and Yang are in equilibrium, the spirit is healed.” The maintenance of normal physiological functions is due to the dynamic equilibrium between Yin and Yang in the human body. Diseases can develop if this equilibrium is disrupted. Modern studies have shown that the microenvironmental states of hypoxia, acidosis, tissue high pressure, and chronic inflammation can induce genetic mutations and trigger abnormal tumor cell proliferation, invasion, metastasis, and tumor angiogenesis [22]. This theory is consistent with the microenvironmental point of view in TCM. The disequilibrium of Yin and Yang as well as the dysregulation of Qi, blood, and fluids can lead to the endogenous production of pathological elements, such as dampness, phlegm, stasis, and toxin, which construct a pathogenic inner environment and finally result in disease development. The individual microenvironment in each patient is the basis for TCM syndrome differentiation during clinical practice. The variation of the microenvironment in each individual patient contributes to the diversity of TCM syndromes [23]. The goal of TCM syndrome differentiation is to determine the appropriate targeted TCM therapy to correct disrupted Qi, blood, and Yin and Yang, as well as to restore the normal equilibrium in the body.

In the present study, we demonstrated that driver gene mutations were associated with TCM syndromes and the cold and heat elements in patients with lung adenocarcinoma. Patients with Qi and Yin deficiency or lung-spleen Qi deficiency were more likely to have a driver gene mutation. Patients with mutant driver genes more often had the cold syndrome element, while those with wild-type driver genes more frequently had the heat syndrome element. Our results were consistent with the findings from other research groups [24–26]. Similarly, Xu et al. have reported that mutant genes were found in lung cancer patients in the following increasing order: phlegm-heat, Qi stagnation and blood stasis, Qi and Yin deficiency, and cold-dampness syndrome; however, their study did not find a statistically significant association between driver gene mutations and the deficiency-excess status [27].

Associations between TCM syndromes and the clinical characteristics of patients with lung cancer have been reported previously, but the results are inconclusive. In the present study, the most frequent TCM syndromes in lung adenocarcinoma patients were Qi and Yin deficiency and lung-spleen Qi deficiency. We did not find any statistically significant association between TCM syndromes and the clinical stage of lung cancer patients. In addition, Gui et al. studied 108 lung cancer patients and found that most of them had Qi deficiency and phlegm dampness syndrome or Qi and Yin deficiency syndrome [28]. Cui et al. also have reported that most patients with non-small-cell lung carcinoma had spleen deficiency and phlegm dampness syndrome or Qi and Yin deficiency [29]. Moreover, Fan et al. classified patients with lung cancer into five TCM syndromes based on the etiology, pathogenesis, and their clinical experience as follows: Yin deficiency and internal heat, Qi and Yin deficiency, spleen deficiency and phlegm damp, Yin and Yang deficiency, and Qi stagnation and blood stasis syndromes; they concluded that most patients with lung cancer had Yin deficiency and internal heat or Qi and Yin deficiency syndrome [30]. Furthermore, Wang et al. studied the clinical stage of 102 patients with lung adenocarcinoma and found that the patients with stage I, II cancer mainly had Qi stagnation and blood stasis or Qi deficiency and phlegm dampness syndrome, whereas the patients with stage III, IV cancer mostly had Qi and Yin deficiency syndrome [21]. Another study has reported that most patients with stage I–III or stage IV lung cancer had lung-spleen Qi deficiency or Qi and Yin deficiency syndrome, respectively [31]. The differences in TCM syndromes reported among these studies might be due to different patient demographics, medical histories, cancer types, and clinical stages, as well as variations in the TCM syndrome differentiation among physicians.

Our study showed associations between TCM syndromes and sex, age, and metastasis of the patients. The female patients with lung adenocarcinoma were more likely to have lung-spleen Qi deficiency or Qi and Yin deficiency, whereas the male patients more frequently had Qi stagnation and blood stasis. In general, the lung cancer patients with Qi stagnation and blood stasis were older than those with lung-spleen Qi deficiency. Additionally, the patients with Yin and Yang deficiency were more likely to develop bone metastasis. These results were consistent with the basic TCM principles as stated in “Suwen: Great Theory of Yin and Yang.” “Yin and Yang are blood and Qi in males and females.” In terms of Yin and Yang, the male is Yang and the female is Yin. Of note, male patients had a high rate of smoking. In TCM, smoking is the evil of heat and toxin, which is prone to eliminate body fluid and condense fluid into phlegm. This can ultimately block the flow of Qi over time and cause blood stasis and internal stagnation to cause excess syndrome. Female patients had a soft body, which could combine with the physiological processes of menstruation and fetal delivery. The onset of disease mostly contributes to deficiency syndrome. Therefore, there is a certain association between TCM syndrome and sex. In addition, “Suwen: Great Theory of Yin and Yang” also said, “at the age of 40 years, Yin and Qi fall in half and daily activities decline. At the age of 50 years, weight increases and the ears and eyes start to blur.” This indicates that Yin and Yang can gradually deplete with aging, which is accompanied by TCM syndrome changes from excess to deficiency. In addition, lung adenocarcinoma usually starts from the pulmonary peripheral areas, which rarely have any clinical symptoms in the early and middle stages of cancer. Most patients have already developed into the late stage of disease at advanced age when they present to the clinic with symptoms. At that time, most elderly patients had deficiency syndrome, especially Qi and Yin deficiency. This could be the reason why most lung cancer patients with Qi and Yin deficiency are older than the patients with other syndromes [32]. The lung is a delicate organ that likes moisture and hates dryness. The evil of cancer and toxin resides in the lung over time, which can consume Yin and finally damage Yang in the lung. All of these can contribute to Yin and Yang deficiency in patients with lung cancer. Our present study provided clinical evidence to correlate both the sex and age with TCM syndromes in patients with lung adenocarcinoma. TCM physicians should consider the sex and age of the patients when determining TCM syndrome differentiation and treatments.

The kidney is the root of Yin and Yang in the body and is the master of bone and marrow production. Toxin and evil penetrate deeply and deplete vital Qi. The evil becomes excessive, and the vitality becomes deficient. Finally, Yin and Yang are dysregulated, which affects the kidney essence and kidney Qi to nourish bones. The cancer toxin can easily take advantage of deficiency and stay in bone, which can explain the association between TCM syndromes and bone metastasis in patients with lung adenocarcinoma.

The strength of this study is that it provides clinical evidence to support the association between TCM syndromes and driver gene mutations in patients with lung adenocarcinoma. This association may have therapeutic indications for selecting the appropriate TCM treatment in individual patients. Similar studies are rarely reported in the English literature. The limitations of the current study are a small sample size and single-center research, which could bring biases to our study results. We were not able to analyze each individual type of genetic mutation due to their small sample sizes. Moreover, TCM syndrome differentiation might have variations in different TCM practices, which could limit generalization of the study results. We also did not study the associations between TCM syndromes and the clinical prognoses of patients.

## 5. Conclusions

In the present study, we demonstrated associations between TCM syndromes and driver gene mutations as well as sex, age, and bone metastasis in patients with lung adenocarcinoma. Our study results provide preliminary evidence of the relationships between macroscopic TCM syndrome differentiation and microscopic pathogenesis changes, which might facilitate the selection of appropriate TCM treatment strategies for patients with lung adenocarcinoma. Future large-scale studies in patients with different ages, sexes, clinical stages, and metastasis locations are warranted.

*M* ± SD, mean ± standard deviation.

## Figures and Tables

**Table 1:** Clinical characteristics of the study participants.

	Driver gene mutations		*P*
	Yes (*n* = 81)	No (*n* = 46)	
Age, years, *M* ± SD	63.4 ± 10.1	63.6 ± 7.7	0.874

Gender, *N* (%)			0.087
Male	33 (40.7)	26 (56.5)	

Smoking history, *N* (%)	30 (37.0)	26 (56.5)	0.034

Clinical stage, *N* (%)			0.963
I-II	5 (6.2)	3 (6.5)	
III	11 (13.6)	7 (15.2)	
IV	65 (80.2)	36 (78.3)	

Metastasis, *N* (%)			0.703
Yes	77 (95.1)	43 (93.5)	

**Table 2:** Associations between traditional Chinese medicine syndromes and driver gene mutations.

Syndrome type	*N*	Driver gene, *n* (%)		*X* ^2^	*P*
		Mutant	Wild type		
Lung-spleen deficiency	32	24 (75.0)	8 (25.0)		
Yin deficiency and internal heat	29	14 (48.3)	15 (51.7)		
Qi and Yin deficiency	39	30 (76.9)	9 (23.1)	11.092	0.026
Qi stagnation and blood stasis	19	10 (52.6)	9 (47.4)		
Yin and Yang deficiency	8	3 (37.5)	5 (62.5)		

**Table 3:** Associations between traditional Chinese medicine syndrome elements and driver gene mutations.

Syndrome elements	*N*	Driver gene, *n* (%)		*X* ^2^	*P*
		Mutant	Wild type		
Cold	66	48 (72.7)	18 (27.3)	4.762	0.029
Heat	61	33 (54.1)	28 (45.9)		
Deficiency	72	48 (66.7)	24 (33.3)		
Excess	29	18 (62.1)	11 (37.9)	0.714	0.700
Mixed deficiency and excess	26	15 (57.7)	11 (42.3)		

**Table 4:** Distributions of different syndromes and elements in patients with or without driver gene mutations.

Syndromes	Mutant type (*n*)						Wild type (*n*)					
	Heat	Cold	Deficiency	Excess	Mixed deficiency and excess	Total	Heat	Cold	Deficiency	Excess	Mixed deficiency and excess	Total
Lung-spleen deficiency	1	23	16	1	7	24	7	1	4	0	4	8
Yin deficiency and internal heat	14	0	8	3	3	14	15	0	8	2	5	15
Qi and Yin deficiency	8	22	21	4	5	30	2	7	0	7	2	9
Qi stagnation and blood stasis	10	0	0	10	0	10	4	5	0	9	0	9
Yin and Yang deficiency	0	3	3	0	0	3	0	5	5	0	0	5

**Table 5:** Associations between traditional Chinese medicine syndromes and clinical characteristics.

Characteristics	*n*	TCM syndrome (*n*)					*P*
		Lung-spleen Qi deficiency	Yin deficiency and internal heat	Qi and Yin deficiency	Qi stagnation and blood stasis	Yin and Yang deficiency	
Sex							
Male	59	11	16	15	15^*∗*^,^#^	2	0.010
Female	68	21	13	24	4	6	

Smoking history							
Yes	56	9	17	19	9	2	0.116
No	71	23	12	20	10	6	

Clinical stage							
I, II	8	1	1	5	1	0	0.595
III	18	5	5	7	1	0	
IV	101	26	23	27	17	8	

^*∗*^Compared with lung-spleen Qi deficiency, *P*=0.002. ^#^Compared with Qi and Yin deficiency, *P*=0.004.

**Table 6:** Associations between traditional Chinese medicine syndromes and age.

Syndrome type	Age, years (mean ± SD)	95% confidence interval	F test	
			*F*	*P*
Lung-spleen deficiency	59.9 ± 9.6 ^*∗*^,^#^	56.5–63.4		
Yin deficiency and internal heat	61.9 ± 8.4	58.7–65.1		
Qi and Yin deficiency	66.2 ± 9.2	63.2–69.2	2.725	0.032
Qi stagnation and blood stasis	66.1 ± 8.7	61.9–70.2		
Yin and Yang deficiency	63.6 ± 7.7	57.2–70.0		

^*∗*^Compared with Qi and Yin deficiency, *P*=0.004.

**Table 7:** Associations between traditional Chinese medicine syndromes and cancer metastasis.

Metastasis		TCM syndrome (*n*)					
		Lung-spleen Qi deficiency	Yin deficiency and internal heat	Qi and Yin deficiency	Qi stagnation and blood stasis	Yin and Yang deficiency	*P*
Lung	Yes	13	11	15	6	4	0.925
	No	19	18	24	13	4	
Brain	Yes	10	5	7	5	4	0.251
	No	22	24	32	14	4	
Bone	Yes	13	15	13	11	7	0.043
	No	19	14	26	8	1	
Liver	Yes	4	4	2	3	0	0.558
	No	28	25	37	16	8	
Pleura	Yes	12	4	8	6	3	0.206
	No	20	25	31	13	5	
Lymph node	Yes	20	22	26	14	4	0.236
	No	12	7	13	5	4	
Multiorgan^*∗*^	Yes	18	16	18	12	5	0.751
	No	14	13	21	7	3	

^*∗*^Multiorgan metastasis refers to metastasis identified in ≥2 organs. ^#^Compared with Qi stagnation and blood stasis, *P*=0.020.

## Data Availability

The data used to support the findings of this study are available from the corresponding author upon request.
